# Efficacy and Safety of Anti-malarial Drugs (Chloroquine and Hydroxy-Chloroquine) in Treatment of COVID-19 Infection: A Systematic Review and Meta-Analysis

**DOI:** 10.3389/fmed.2020.00482

**Published:** 2020-07-29

**Authors:** Rashmi Ranjan Das, Nishant Jaiswal, Nishanth Dev, Nikita Jaiswal, Sushree Samiksha Naik, Jhuma Sankar

**Affiliations:** ^1^Department of Pediatrics, AIIMS Bhubaneswar, Bhubaneswar, India; ^2^Evidence Based Health Informatics Unit, Department of Telemedicine, PGIMER, Chandigarh, India; ^3^Department of Medicine, VMMC, Safdarjung Hospital, New Delhi, India; ^4^Department of Microbiology, MM Institute of Medical Sciences and Research, MMU (Deemed to be University), Ambala, India; ^5^Department of Obstetrics and Gynecology, Capital Hospital, Bhubaneswar, India; ^6^Department of Pediatrics, AIIMS New Delhi, New Delhi, India

**Keywords:** aminoquinoline, azithromycin, SARS-CoV-2, evidence-based medicine, COVID-19, mortality, Chloroquine, Hydroxychloroquine

## Abstract

**Background:** Anti-malarial drugs inhibit coronaviruses *in-vitro*. Few published studies have evaluated the safety and efficacy of these drugs in the treatment of COVID-19 infection.

**Materials and Methods:** This is a systematic review and meta-analysis of clinical trials and observational studies. Major database searches were carried out up until June 5, 2020. Participants admitted with RT-PCR-confirmed SARS Cov-2 (COVID-19) infection were included. The “Intervention group” received anti-malarial drugs with or without other drugs (Azithromycin) administered as an adjunct to the standard treatment/care. The “Control group” received treatment except anti-malarial drugs. The primary outcome is “all-cause mortality.” Secondary outcome measures were effects on clinical and laboratory parameters and adverse events.

**Results:** Of 3,472 citations, 17 (six clinical trials and 11 observational studies) studies provided data of 8,071 participants. Compared to the control, Hydroxy-chloroquine (HCQ) has no significant effect on mortality [(OR 0.87; 95% CI 0.46–1.64); eight observational studies; *N* = 5,944]. Data from a single, small non-randomized trial (*N* = 42) also reached a similar conclusion (OR 1.94; 95% CI 0.07–50.57; *p* = 0.69). Compared to the control, HCQ plus Azithromycin (AZM) significantly increased mortality [(OR 2.84; 95% CI 2.19–3.69); four observational studies; *N* = 2,310]. Compared to the control, risk of any adverse event was significantly increased in HCQ group [(OR 3.35; 95% CI 1.58–7.13); four clinical trials; *N* = 263]. Compared to control, risk of adverse cardiac events (abnormal ECG, arrhythmia, or QT prolongation) were not significantly increased in HCQ group (but significantly increased in the HCQ plus AZM group). The GRADE evidence generated for all the outcomes was of “very low-quality.”

**Conclusions:** As very low quality evidence suggests an increased risk of mortality and adverse event with HCQ plus Azithromycin combination (not HCQ alone), caution should be exercised while prescribing this combination for treatment of hospitalized adults with COVID-19 infection. Good quality, multi-centric RCTs (including both hospitalized and non-hospitalized patients) are required for any firm recommendation to be made during the ongoing pandemic.

**OSF Protocol Registration Link:** https://osf.io/6zxsu.

## Introduction

COVID-19, also known as severe acute respiratory syndrome coronavirus 2 (SARS-CoV-2), emerged in Wuhan, China, in late 2019. It is a highly contagious disease with a global average mortality rate of 4.6% (1). There have been ongoing efforts to develop effective treatment modalities for this dreaded pandemic. Currently, no specific therapies against SARS-CoV-2 infection exist, and a series of therapeutic agents (e.g., antiviral agents, antibiotics, immune-modulators, inhaled nitric oxide, and convalescent plasma) have been repurposed with negative to inconclusive evidence available (2). There has been an increased interest in two existing anti-malarial drugs belonging to amino-quinoline group (Chloroquine and Hydroxy-chloroquine) to treat COVID-19. This is because of the inhibitory effects of these two drugs on other coronaviruses, such as SARS-CoV-1 (2, 3). The plausible mechanism of actions includes inhibition of angiotensin converting enzyme 2 (ACE-2) present on the cell surface for virus entry (by reduction of glycosylation in the enzyme) (4, 5), inhibition of release of viral particles into intra-cellular space (6, 7), and an anti-inflammatory effect (inhibition of interleukin-6, the tumor necrosis factor, the aberrant interferon, and other pro-inflammatory cytokines that cause lung injury leading to acute respiratory distress syndrome) (6, 8). Chloroquine and Hydroxy-chloroquine (HCQ) are both cost-effective and considered safe as per their approved indications. Compared to Chloroquine (CQ), Hydroxy-chloroquine (HCQ) is more soluble and less toxic and is considered safer (9, 10). It has to be kept in mind that these drugs are not entirely safe because of the risk of some serious side-effects (e.g., neuro-psychiatric, retinal, cardiac, and hypoglycemia), and there have been reports of toxicities in people who are self-medicating (11, 12).

There have been published studies evaluating the safety and/or efficacy of these agents (alone or in combination) compared to a control arm or parallel intervention, to treat patients with COVID-19 (13–32). However, the results have been contradictory. Earlier published rapid systematic reviews have concluded the role of anti-malarial drugs in patients with COVID-19 is still uncertain, and its routine use should not be recommended until more evidence is available from ongoing studies (33, 34). However, these systematic reviews neither included larger observational studies and randomized clinical trials (RCTs) published recently nor provided quality (GRADE) of evidence in a more systematic manner. In addition, findings from an ORCHID (Outcomes Related to COVID-19 treated with hydrox-ychloroquine among In-patients with symptomatic Disease) study have shown that HCQ neither harms nor benefits patients with COVID-19 infection (35). The present systematic review is an endeavor in this direction to synthesize the available evidences to inform clinical practice and guide the international agencies to formulate recommendations.

## Materials and Methods

This systematic review protocol is registered at the Open Science Forum (OSF) registration link: https://osf.io/6zxsu.

### Types of Studies

Both clinical trials (randomized, quasi- randomized, and non-randomized) and observational studies comparing anti-malarial drugs (Chloroquine and Hydroxy-chloroquine) alone or in combination with other drugs vs. a control (standard of care) or other treatment were included. As a majority of the studies were published on pre-print servers (for rapid dissemination of knowledge) prior to publication in peer-reviewed journals, we planned to include these studies in the present meta-analysis after taking permission from the study authors.

### Types of Participants

Children of 12–18 years of age and adults with RT-PCR-confirmed SARS Cov-2 (COVID-19) cases treated in the hospital were included. Exclusion criteria were an allergy to anti-malarial drugs [Chloroquine (CQ) and Hydroxy-chloroquine (HCQ)], retinopathy, hearing loss, and severe neuro-psychiatric diseases.

### Types of Interventions

- Interventions included anti-malarial drugs (CQ and HCQ) provided in various formulations and dose schedules. Based on a previous study, the following dose schedules were considered: HCQ—a loading dose of 400 mg twice daily (BID) followed by a maintenance dose of 200 mg BID for 4 days; and CQ-−500 mg BID for 5 days (9). The intervention was administered as an adjunct to other treatment modalities [including Azithormycin (AZM)] to patients infected with SARS Cov-2 (COVID-19). Those in the control group received supportive treatments without CQ/HCQ. We also included trials comparing different doses (high dose vs. low-dose of anti-malarial drugs) to provide more information and urgent dissemination of knowledge during the current pandemic.
- Supportive and additional treatment included various methods. In hospitalized cases, it varied from bed rest, nebulization, and oxygen inhalation to invasive respiratory support (mechanical ventilation) and maintenance of vital parameters. In addition, additional treatment during the current pandemic included antibiotics, non-specific anti-viral drugs [Remdesivir, Lopinavir/Ritonavir, IFN-α/β, Umifenovir [Arbidol], Entecavir, Ribavirin, and/or Oseltamivir], Immuno-modulators (Immunoglobulin, Tocilizumab, and Sarilumab), steroids, and NSAIDs (including Aspirin). There is evidence that non-specific antiviral drugs may not benefit patients with Covid-19, though Remdesivir and immune modulators may have some role in severe or critical cases (36, 37).

### Types of Outcome Measures

#### Primary

1. All-cause mortality: patients with Covid-19 dying from any cause.

#### Secondary

1. Time to clinical recovery: time taken for normalization of temperature, respiratory distress, and relief of cough or no cough for 72 h
2. Proportion of patients with clinical recovery: proportions of patients with normalization of temperature, respiratory distress, and relief of cough or no cough for 72 h
3. Proportion of patients requiring escalation of respiratory support (including mechanical ventilation) or requiring ICU transfer: escalation of respiratory support defined as progressive change in the requirement of respiratory support to maintain normal oxygen saturation (SpO_2_) and vital parameters
4. Proportion of patients developing severe disease: proportions of patients developing severe disease as defined as per the National Institute of Health (NIH) COVID-19 Treatment Guidelines (37)
5. Duration of hospitalization: the time from admission (days) to either discharge or death
6. Duration of ICU stay: the time from admission (days) to ICU to death or transfer back to non-critical areas
7. Time to negative PCR results for COVID-19: the time taken for two consecutive negative reports of a positive patient
8. Proportion of patients with negative PCR results for COVID-19 after day 3, 5, 7, 10, 14, 21, and 28: proportions of patients with two consecutive negative reports after a positive report
9. Proportion of patients with improved radiological features after day 3, 5, 7, 10, 14, 21, and 28: proportions of patients with improvement noted in either chest X ray or CT scan of chest compared to that done at baseline
10. Effect on hematological parameters (including inflammatory markers): these include the blood parameters (complete blood count, differential counts, and platelet count), acute phase reactants (ESR, CRP, and pro-calcitonin), and inflammatory markers (IL-6, TNF-α, etc.)
11. Adverse events: developing secondary to the use of anti-malarial drugs alone or in combination with other drugs.

### Search Methodology

The following major databases were searched systematically from 1970 till June 5, 2020: Cochrane Central Register of Controlled Trials (CENTRAL), PubMed/MEDLINE, Google Scholar, and EMBASE (Appendix 1). We also searched the Pre-print servers (medRxiv, bioRxiv, OSF pre-prints, Pre-prints.org) till June 5, 2020. The PubMed/Medline search strategy used the various MeSH and free text terms for “novel corona virus,” “COVID 19,” “Hydroxychloroquine,” and “Chloroquine” combined using the Boolean operators. No language restrictions were applied. Three reviewers (RRD, NJ, and ND) reviewed the search results to identify relevant studies.

### Data Extraction

Data extraction was done using a data extraction form that was designed and pilot tested a priori. Three authors (NJ, ND, and SSN) independently extracted the following information from each study: author; year; location (country); study design (clinical trial or observational study); setting (hospital or community); method of recruitment; inclusion criteria; unit of analysis; allocation ratio In case of RCT); risk of bias; participants (age, sex, sample size, and disease severity); intervention (dosage, duration, frequency, and co-intervention if any); outcomes (outcome definition, valid unit of measurement, time points of collection and reporting); loss to follow-up; and miscellaneous (key conclusions, references to other relevant trials, and additional data required).

### Assessment of Risk of Bias in the Included Studies

Two review authors independently (NJ and SSN) assessed the methodological quality of the selected trials by using methodological quality assessment forms and the criteria outlined in the Cochrane Handbook for Systematic Reviews of Interventions (38). Quality assessment was undertaken using the Newcastle Ottawa Scale (NOS) for observational studies. This scale assesses the quality under three major headings, namely, selection of the studies (representativeness and the exposure assessment/control selection), comparability (adjustment for main/additional confounders), and outcome/exposure (adequacy of outcome measured, exposure measured vs. self-report) (39). Quality assessment was undertaken using the ROBINS-I tool for non-randomized trials (40). Any disagreements between the two review authors were resolved through discussion with a third author (JS).

### Dealing With Missing Data

We described missing data, including dropouts in included studies. Differential dropout rates can lead to biased estimates of the effect size, and bias may arise if the reasons for dropping out differ across groups. We reported reasons participants dropped out of studies as mentioned by the authors. If data were missing, or if reasons for dropping out were not reported, we contacted the authors for further information.

### Data Synthesis

Data were analyzed using Review Manager (RevMan) V.5.1 (The Nordic Cochrane Center, The Cochrane Collaboration, Copenhagen, Denmark) (41). The data from various studies were pooled and expressed as mean difference (MD) with 95% confidence interval (CI) in case of continuous data, and odds ratio (OR) with 95% CI in case of categorical data. Where data were expressed as a median (IQR), we calculated the mean and SD by the statistical formula described previously (42). The primary pooled analysis of all the reports was conducted using the Generic Inverse Variance method using random effects weighting (43), where the log RRs for cohort studies or log ORs for case–control studies were weighted by the inverse of the variance to obtain a pooled RR estimate. Since nested case-cohort and nested case–control studies are temporally prospective, we analyzed data from these studies with the prospective studies. A *p* < 0.05 was considered statistically significant. Inter-study heterogeneity was assessed by Cochrane's Q (χ^2^ *p* < 0.10) and quantified by *I*^2^. An *I*^2^ ≥ 50% indicated “substantial” heterogeneity and ≥75% indicated “considerable” heterogeneity (44). The cause of substantial and considerable heterogeneity was explored, and sensitivity and/or sub-group analyses were carried out.

### Publication Bias

To evaluate for any possible publication bias, we constructed the funnel plot from primary outcome data (45).

### Grade of Evidence

To assess the quality of evidence we used GRADE Profiler software (V.3.2) (46, 47). The software uses five parameters for rating the quality of evidence. The parameters used were limitations to design of randomized controlled trials, inconsistency of results or unexplained heterogeneity, indirectness of evidence, imprecision of results, and publication bias. The rating was determined as no, serious, or very serious limitations.

## Results

### Description of Studies

**Figure 1:**
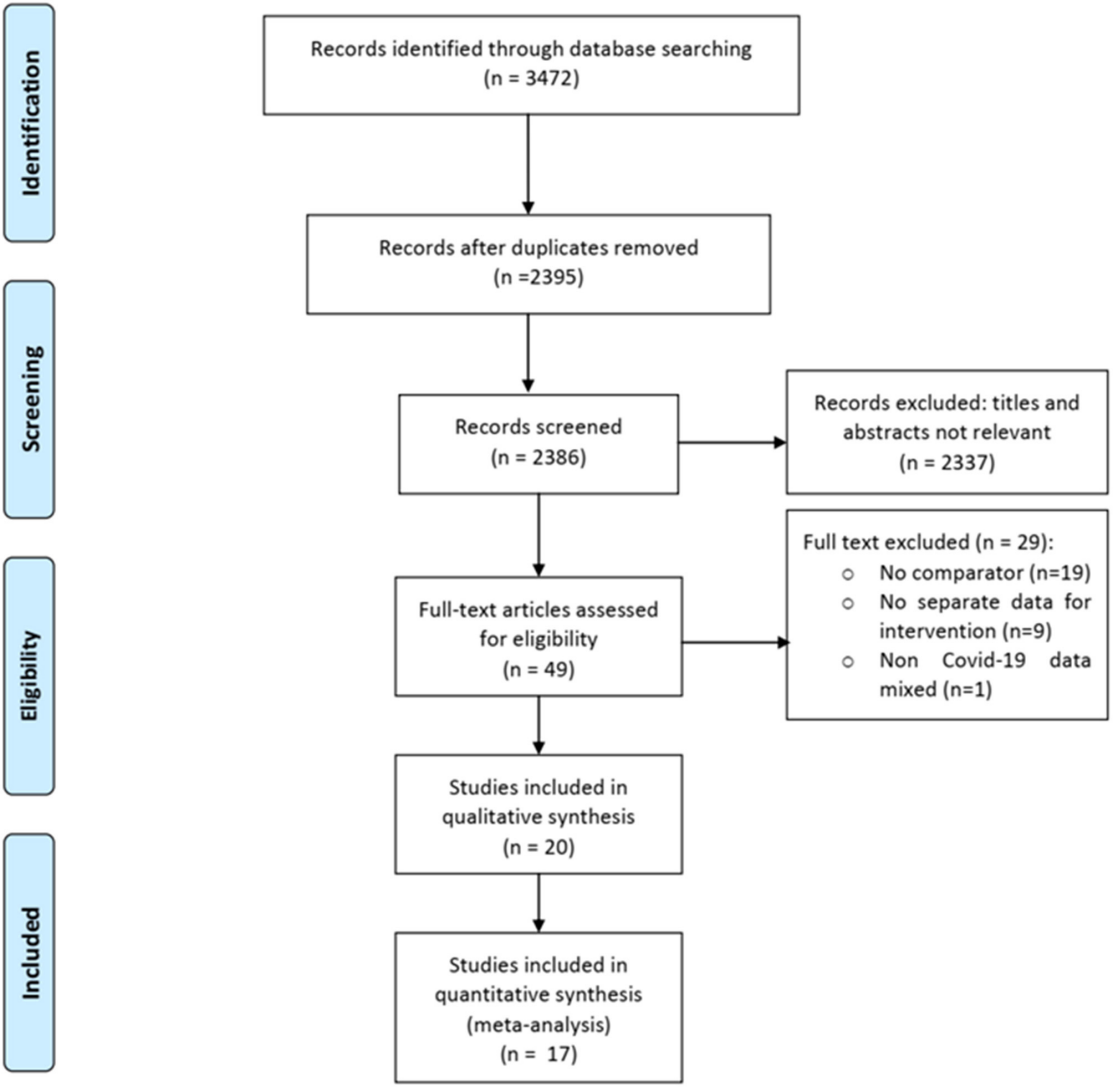
PRISMA flow diagram.

Of 3,472 total citations retrieved, the full texts of 49 papers were assessed for eligibility, and 29 were excluded for various reasons (Figure 1). Of the remaining 20 eligible studies, 14 were published in peer-reviewed journals (13–29) and six in pre-print servers (not peer-reviewed) (17, 28–32). We contacted the authors of these six studies to give us their permission to use their data in the meta-analysis, but only three authors gave their permission (17, 28, 29). We therefore included the data of three studies in the meta-analysis and described the characteristics of the remaining three studies using a separate table (Supplementary Table 1). Finally, we were able to conduct a meta-analysis of a total of 17 studies (six clinical trials and 11 observational studies) including 8,071 patients (Adults = 8,041; Adolescents = 30) (Table 1). Twenty-nine studies were excluded for the following reasons: 19 were case series (without having a control/comparator that is inclusion criteria of present review), nine studies mentioned about intervention but did not provide outcome data for them separately, and one study reported use of anti-malarial drugs with or without AZM in rheumatoid arthritis (RA) patients for non-RA indications (including viral and other infections).

**Table 1:** Characteristics of included studies.

**Study author, Country (Reference)**	Sample size (*N*), additional inclusion criteria^*^	**Age and sex of participants**	**Illness severity of participants**	**Intervention group (dose schedule)**	**Time from onset of symptom to treatment (d)**	**Supportive and additional treatment used**	**Additional comments**
**CLINICAL TRIALS (RANDOMIZED AND NON-RANDOMIZED)**							
Gautret et al. France (single center) (13)	*N*: 36 (HCQ = 14; HCQ+AZM = 6; Control =16). Additional inclusion criteria: None.	Age (yr): >12 yr (HCQ = 51.2 ± 18.7; Control = 37.3 ± 24). Male: 41.7%.	All severity included. Asymptomatic: 16.7% URTI: 61.1% LRTI: 22.2%.	HCQ: 600 mg/d (200 mg TID) for 10 days. HCQ+AZM: AZM 500 mg on day 1 followed by 250 mg OD for 4 days in addition to HCQ.	Mean (SD): 4.1 (2.6) in HCQ group, and 3.9 (2.8) in Control group.	Symptomatic and antibiotics.	HCQ group recruited in one center and control group in another. Control group included those refused intervention or were not eligible for it. Attrition rate 23% in HCQ group. Funded study. There were protocol deviations.
Chen et al. China (single center) (14)	*N*: 30 (HCQ = 15; Control = 15). Additional inclusion criteria: None.	Age (yr): >18 yr (HCQ = 50.5 ± 3.8; Control = 46.7 ± 3.6). Male: 70%.	Severe illness or other measures of severity not defined.	HCQ: 400 mg/d (OD) for 5 days.	Not mentioned.	Respiratory support and others (anti-virals, IFN-α, nebulisation, and antibiotics). Arbidol (Umifenovir): HCQ group (80%), Control group (67%). Lopinavir/Ritonavir: HCQ group (0%), Control group (13%).	Underlying co-morbidities: hypertension (27%), diabetes (7%), and chronic obstructive lung disease (3.5%). Started enrolment 1 day prior to trial registration. Funded study.
Tang et al. China (multi-center) (15)	*N*: 150 (HCQ = 75; Control = 75). Additional inclusion criteria: A Chest CT scan needed before randomization.	Age (yr): >18 yr (HCQ = 48.0 ± 14.1; Control = 44.1 ± 15.0). Male: 54.7%.	Mild: 14.7% Moderate: 84% Severe: 1.3%.	HCQ: 1,200 mg/d for 3 days followed by 800 mg/d for the remaining days (total treatment duration: 2 weeks for mild/moderate and 3 weeks for severe cases).	Mean: 16.6 (HCQ started within 24 h of randomization).	Respiratory support. ntibiotics (39%), anti-virals (Lopinavir/Ritonavir, Arbidol, Ribavirin, and/or Oseltamivir), and Steroid (7%).	Trial stopped early (intended to enroll 360 patients-−180 in each arm). Underlying co-morbidities (30%): diabetes (14%), hypertension (6%), and others (20.7%). Funded study. Shanghai Pharma donated HCQ.
Huang et al. China (single center) (16)	*N*: 22 (CQ = 10; Control = 12). Additional inclusion criteria: None.	Age (yr): >18 yr (CQ [median, IQR] = 41.5 [33.8–50]; Control [median, IQR] = 53 [41.8–63.5]). Male: 59.1%.	Moderate: 64% Severe: 36%.	CQ: 1,000 mg/d (500 mg BID) for 10 days. Lopinavir (400 mg)/Ritonavir (100 mg): BID for 10 days in the control group.	Median: 2.5 in CQ group, and 6.5 in Control group.	Respiratory support, antibiotics, anti-virals, and steroid.	Underlying co-morbidities: hypertension (18.2%), diabetes (9.1%), smoking (9.1%), and cerebro-vascular disease (4.5%). No protocol deviation. Funding status not mentioned.
Chen et al. China (single center) (17)	*N*: 62 (CQ = 31; Control = 31). Additional inclusion criteria: Chest CT with pneumonia; SaO_2_/SPO_2_ ratio > 93% or PaO_2_/FIO_2_ ratio > 300 mmHg (mild illness).	Age (yr): >18 yr (HCQ = 44.1 ± 16.1; Control = 45.2 ± 14.7). Male: 46.8%.	Mild: 100%	HCQ: 400 mg/d (200 mg BID) for 5 days.	Both groups had fever and cough 1 day before starting of intervention (intervention started within 48 h)	Oxygen therapy, antiviral agents, antibacterial agents, and Immunoglobulin, with or without Corticosteroids.	No information on underlying co-morbidities. Significant deviation from registered protocol. Funded study. Shanghai Pharma provided the HCQ tablets.
Borba et al. Brazil (single center) (18)	*N*: 81 (CQ high-dose = 41; CQ low-dose = 40). Additional inclusion criteria: RR >24/min and/or HR >125 bpm and/or SpO_2_ <90% in ambient air and/or shock.	Age (yr): >18 yr (CQ high-dose = 54.7 ± 13.7; CQ low-dose = 47.4 ± 13.3). Male: 75.3%.	Severe: 89% (33% were critical)	High-dose CQ: 600 mg BID for 10 days (total dose 12 g). Low-dose CQ: 450 mg BID on day 1 followed by OD for 4 days (total dose 2.7 g).		Ceftriaxone (7 days) plus azithromycin (5 days) in all cases, and Oseltamivir (5 days) in 87% cases.	Co-morbidities: hypertension (45.5%), alcohol disorder (27.5%), and diabetes (25.5%). Older and more heart disease (high-dose = 17.9%, low-dose = 0) in the high-dose group. Funded study.
**OBSERVATIONAL STUDIES**							
Mahévas et al. France (multi- center) (19)	*N*: 181 (HCQ = 84; Control = 97). Additional inclusion criteria: requiring oxygen by mask or nasal prongs (WHO progression scores of 5).	Age (yr): >18 yr (HCQ [median, IQR] = 59 [48–67]; Control [median, IQR] = 62 [55–69]. Male: 72%.	Severe: 100%.	HCQ: 600 mg/d	Median: 7 (HCQ started within 24 h of admission except in 8 cases).	Respiratory support, Azithromycin (HCQ = 18%, Control = 29%); Amoxicillin and Clavulanic acid (HCQ = 52%, Control = 28%). No patient received anti-viral drugs or anti-inflammatory drugs.	Co-morbidities: cardio-vascular disease (55%), obesity (26%), immunosuppression (12%), chronic respiratory disease (11%), diabetes (9%), and chronic kidney disease (5%). Virological cure (repeat PCR) not checked. Non-funded study.
Geleris et al. USA (single center) (20)	*N*: 1,376 (CQ = 811; Control = 565). Additional inclusion criteria: None.	Age (yr): >18 yr [Majority were ≥60 years of age (60.5%)]. Male: 56.8%.	Severe illness or other measures of severity not defined (HCQ group were more severely ill than control group).	HCQ: 600 mg BID on day 1 followed by 400 mg OD for 4 days.	Not mentioned (in 86% cases, HCQ started within 48 h of admission).	Respiratory support, antibiotics (66.1%), Azithromycin (44.5%), Tocilizumab (5.1%), Remdesivir (2%), Sarilumab (2.2%), and Corticosteroids (19.8%).	Co-morbidities: diabetes (35.7%), hypertension (31.7%), chronic lung disease (18.2%), chronic kidney disease (17.3%), cancer (12.8%), smoking (11.4%), and transplant/HIV/immunosuppression (4.2%).
Rosenberg et al. USA (multi-center) (21)	*N*: 1,438 (HCQ = 271; HCQ+AZM = 735; AZM = 211; Control = 221). Additional inclusion criteria: None.	Age (yr) (median): Children and Adults (HCQ = 65.5; HCQ+AZM = 61.4; Control = 64). Male: 59.7%.	All severity included (HCQ group: 30% critically ill; Control group: 10% critically ill). Only HCQ group had the highest levels of chronic lung disease (25.1%) and cardiovascular conditions (36.5%). Obese, diabetes, dementia Black or Hispanic patients, clinically severity score and abnormal radiological findings were significantly more in HCQ group.	HCQ: 200–600 mg in OD or BID schedule (variably used).	Median: three in the HCQ group, two in the HCQ+AZM group, and four in the Control group (HCQ started within 48 h of admission).	Respiratory support, Aspirin (19.8%), and NSAIDs (3.6%).	Included 25 children. Co-morbidities: diabetes (35%), obesity (30.4%), cardio-vascular disease (30.4%), chronic lung disease (18%), smoking (17.4%), kidney disease (13%), dementia (6.5%), and cancer (3.8%). Patients entered the ICU/mechanical ventilated, often with HCQ and AZM initiation, rendered these outcomes unsuitable for efficacy analyses. Adverse events were collected, potentially before drug initiation. Conflict of interest unclear (spouse of one author received grant from Gilead foundation). Funded study.
Saleh et al. USA (single center) (22)	*N*: 201 (CQ/HCQ = 82; CQ/HCQ+AZM = 119). Additional inclusion criteria: None.	Age (yr): >18 yr (mean ± SD = 58.5 ± 9.1). Male: 57.2%.	Severe illness or other measures of severity not defined.	CQ/HCQ: CQ 500 mg BID on day 1 followed by OD for 4 days; HCQ 400 mg BID on day 1 followed by 200 mg BID for 4 days (total 5 days. HCQ+AZM: AZM 500 mg OD for 5 days in addition to above.	Not mentioned.	Respiratory support.	Co-morbidities: hypertension (60.2%), hyperlipidemia (41.8%), diabetes (32.3%), chronic lung diseases (14.9%), coronary artery disease (11.4%), heart failure (7.5%), atrial fibrillation (7%), and chronic kidney disease (5%). No virological outcome studied. Non-funded study.
Yu et al. China (Single- center) (23)	*N*: 550 (HCQ = 48; Control = 502). Additional inclusion criteria: CT chest suggestive and critically ill (corresponding to a WHO progression score of 5).	Age (yr) [median (IQR)]: >18 yr [HCQ = 68 (60–75); Control = 68 (59–77)]. Male: 62.5%.	Critically ill (100%).	HCQ: 400 mg/d (200 mg BID for 7–10 days).	Median (IQR): 10 (3–13) after admission.	Respiratory support. antivirals (Lopinavir/Ritonavir, Entecavir hydrate, or Ribavirin), IVIg, antibiotics, and Interferon (no Interferon in HCQ group).	Co-morbidities were: hypertension (45.8%), diabetes (17.1%), coronary heart disease (10.7%), and COPD (2.9%). Funded study.
Huang et al. China (Multi- center) (24)	*N*: 373 (CQ = 197; Control = 176). Additional inclusion criteria: None.	Age (yr) [median (IQR)]: >18 yr [CQ = 43 (33–55); Control = 47.5 (35.8–56)]. Male: 46.9%.	Mid: 3.8% Moderate: 91.4% Severe: 4.8%.	CQ: 500–1,000 mg/d (OD or BID) for 10 days.	Median (IQR): 7 (3–10.8) after admission (Guangdong province). Median (IQR): 19 (17–124.5) after admission (Hubei province).	Respiratory support. Only Control group received following treatment: antivirals (Arbidol, Lopinavir/Ritonavir), Chinese traditional medicine, and Interferon.	Co-morbidities were: hypertension (6.4%) and diabetes (2.4%). Funded study.
Magagnoli et al. USA (single center) (25)	*N*: 807 (HCQ = 198; HCQ+AZM = 214; Control = 395). Additional inclusion criteria: Availability of data on body mass index, vital parameters.	Age (yr) [median (IQR)]: >18 yr [HCQ = 71 (62–76.8); HCQ+AZM = 68 (59–74); Control = 70 (59–77)]. Male: 95.7%	All severity included (no severity subgroups mentioned).	HCQ [median (IQR) daily dose]: 400 (400–480) mg in HCQ group, and 422.2 (400–480) mg in HC+AZM group for median (IQR duration of 5 (3–6) d.	Not mentioned (HCQ and AZM started within 24 h).	Respiratory support, and Azithromycin (23% in control group only).	Co-morbidities: Diabetes mellitus (66.2%), cardio-vascular disease (42.9%), renal disease (25%), chronic pulmonary disease (19.6%), malignancy (18%), hyper-lipidemia (15.8%), cerebro-vascular diseases (15%), smoking (14.1%), liver disease (9.2%), dementia (8.4%), asthma (6%), and HIV/AIDS (2.4%). There were significant differences among the three groups in baseline demographic characteristics, selected vital signs, laboratory tests, prescription drug use, and co-morbidities.
Ayerbe et al. Spain (multi- center) (26)	Number: 2,019 (HCQ = 1,857; Control = 162). Additional inclusion criteria: None.	Age (yr): >18 yr (HCQ = 67.11 ± 15.51; Control = 73.47 ± 16.22) Male: 57.3%.	All severity included (no severity subgroups mentioned).	HCQ: dose and schedule not mentioned.	Not mentioned.	Respiratory support, anti-virals (Lopinavir/Ritonavir), Tocilizumab, Steroids, Heparin, and Oseltamivir.	No information on underlying co-morbidities. Funded study.
Kuderer et al. USA, Canada, and Spain (multi- center) (27)	Number: 756 (HCQ = 89; HCQ+AZM = 181; Control = 486). Additional inclusion criteria: underlying malignancy.	Age (yr) [median (IQR)]: >18 yr [66 (57–76)]. Male: 50%.	All severity included (no severity subgroups mentioned).	HCQ: dose and schedule not mentioned.	Not mentioned.	Not mentioned except for the specific treatment of malignancy	Co-morbidities: Malignancy (100%), and obesity (19%). Funded study. HCQ+AZM was given to patients with severe illness.
Mallat et al. UAE (single center) (28)	N: 34 (HCQ = 23; Control = 11). Additional inclusion criteria: None.	Age (yr): [median (IQR)]: >18 yr [HCQ = 33 (31 – 48); Control = 41 (30–55)]. Male: 73.5%.	Mild and moderate (100%).	HCQ: 800 mg/d (400 mg BID) on day 1 400 mg/d for 10 days.	Median: 4 (HCQ started within 24 h).	Respiratory support. Others not mentioned.	Co-morbidities: hypertension (14.7%), asthma (8.8%), diabetes (5.9%), heart disease (2.9%), renal disease (2.9%), and immunosuppressant use (2.9%). Co-morbidities and D-dimer levels were significantly higher in the non-HCQ group.
Membrillo et al. Spain (single center) (29)	Number: 166 (HCQ = 123; Control = 43). Additional inclusion criteria: bilateral pneumonia with clinical picture compatible with COVID-19.	Age (yr): >18 yr (HCQ = 61.5 ± 16.2; Control = 68.7 ± 18.8). Male: 62%.	Mild: 50% Moderate: 29% Severe: 21%.	HCQ: 1,200 mg (800 mg + 400 mg) loading dose on day 1 followed by 400 mg OD.	Median: 7 in HCQ group (started within 24 h).	Respiratory support, anti-virals (Lopinavir/Ritonavir), IFN-β), and/or anti-inflammatory drugs (steroids and/or tocilizumab).	Co-morbidities: hypertension (42.8%), dyslipidemia (34.3%), heart disease (22.3%), diabetes (17.5%), cancer (13.9%), and pulmonary disease (14.4%).

*HCQ, Hydroxychloroquine; CQ, Chloroquine; AZM, Azithromycin; RT-PCR, Reverse transcription polymerase chain reaction; MV, Mechanical ventilation; COPD, Chronic obstructive pulmonary disease; URTI, Upper respiratory tract infection; LRTI, Lower respiratory tract infection; ARDS, Acute respiratory distress syndrome; IQR, Inter-quartile range; ICU, Intensive care unit; WHO, World health organization; OD, Once daily; BID, Twice daily; NSAIDs, Non-steroidal anti-inflammatory drugs; IVIg, intravenous immunoglobulin*.

* *Additional inclusion criteria, any additional features besides RT-PCR positive SARS-CoV-2*.

Of 17 published peer-reviewed studies included, six clinical trials provide data of 381 patients, and the 11 observational studies provided data of 8,071 patients. A total of 4,009 patients received HCQ or CQ (clinical trials = 226, observational studies = 3,783), and 1,255 received a combination of HCQ plus Azithromycin (clinical trials = 06, observational studies = 1,249). The studies were conducted in following countries: USA (five studies, 3,985 patients), Spain (two studies, 2,185 patients), China (four studies, 752 patients), France (two studies, 217 patients), Brazil (one study, 81 patients), and the UAE (one study, 34 patients). One trial compared high vs. low-dose of Chloroquine (18). One clinical trial (13) and six observational studies (19–22, 25, 27); each had three arms of comparison (HCQ, HCQ+AZM, and Control). Two studies included data on adolescents (<18 years) (13, 21). Two studies used Azithromycin but did not provide separate outcome data for both the groups (19, 20). Of the six clinical trials, three were described as double blinded, two were open label, and one was a non-randomized trial.

As shown in Table 1, the age of included participants, severity of illness, dose schedule, and timing of administration of intervention (HCQ/CQ) varied widely among the studies. Majority of the participants in the clinical trials were ≤ 50 yr of age, whereas, majority of the participants in the observational studies (except one) were ≥60 yr of age. Around 72% of participants in the clinical trials were having mild and moderate illness, whereas <40% of the participants in the observational studies were having mild and moderate illness. One study included only cancer patients (27). The dose of CQ was nearly uniform (except one RCT comparing high and low-dose) with duration varying from 5 to 10 days. The dose of HCQ varied widely with the lowest dose being 200 mg/d−1,200 mg on day 1 followed by variable doses for variable period (sometime till discharge/death). Two studies did not provide any information on dose schedule of HCQ (26, 27). The median time from onset of symptom to admission or treatment initiation was ≤ 8 days in all but two studies (one RCT has 17 days, and one observational study has 10 days). Two studies did not provide any information on the timing of initiation of HCQ (26, 27). Except one study (17), no other study was able to start the intervention (HCQ/CQ) in the early phase of illness (within 48 h of symptom onset), which is regarded as the golden window for antiviral treatment (e.g., in influenza) (48).

### Risk of Bias in Included Studies

The details have been provided in Appendix 2. Except two trials (17, 18), others had low to high-risk of bias in different domains. One non-randomized trial had a serious risk of bias overall (13). Of the 11 observational studies, five were at a high risk of bias for selection of cases (22, 23, 25, 28, 29). Except for one study (27), the remaining 10 studies were at a high risk of bias for selection of controls and a low risk of bias for the exposure parameters.

### Effect of Interventions

#### Primary Outcomes (All-Cause Mortality)

##### HCQ vs. control

**Figure 2:**
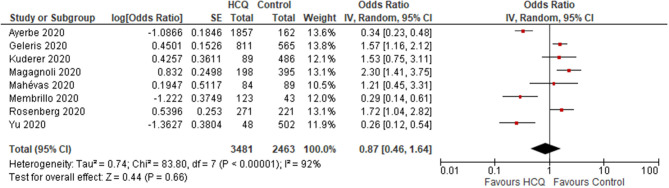
All-cause mortality (HCQ vs. control; observational studies).

- overall results: Three trials reported no mortality in any of the groups. One Non-RCT (*N* = 42) found no significant difference in the mortality rate between HCQ and control group (OR 1.94; 95% CI 0.07–50.57; *p* = 0.69) (13) (Supplementary Figure 1). Eight observational studies (*N* = 5,944) reported mortality rate, and found no significant difference between the HCQ and control group (OR 0.87; 95% CI 0.46–1.64; *p* = 0.66; *I*^2^ = 92%) (Figure 2) (19–21, 23, 25–27, 29).
- Subgroup analysis (data from observational studies): Mortality rate was found to be significantly increased in the HCQ group in the studies from USA (OR 1.71; 95% CI 1.38–2.13; *p* < 0.001; *I*^2^ = 0%; *N* = 3,036) (20, 21, 25, 27), whereas a significantly decreased mortality rate was found in the studies conducted outside USA (*N* = 2,908) population (OR 0.38; 95% CI 0.23–0.63; *p* < 0.001; *I* ^2^ = 56%) (19, 23, 26, 29). The heterogeneity was not significant once we separated studies conducted in USA vs. outside USA. Two studies (20, 23) compared mortality rate in participants aged ≤ 60 vs. >60 yr and found significantly increased risk in those >60 yr age [data provided as hazard ratio [not raw data]]. One study used HCQ after 48 h of admission and two studies had no information on timing; when these two studies were omitted, no difference in mortality was found (OR 1.24; 95% CI 0.7–2.18; *p* = 0.46; *I*^2^ = 82%) (23, 26, 27). When studies with median time from onset of symptom to admission or treatment initiation of >8 days were excluded, and no significant difference was found (OR 1.24; 95% CI 0.7–2.18; *p* = 0.46; *I*^2^ = 82%). We could not carry out subgroup analyses of mortality rate in participants with and without co-morbidity, as these data were not provided separately by the included studies. When studies that did not follow the recommended dose schedule of HCQ/CQ were excluded, still no significant difference was found (OR 0.83; 95% CI 0.36–1.88; *p* = 0.65; *I*^2^ = 90%).

##### HCQ plus azithromycin (AZM)

**Figure 3:**
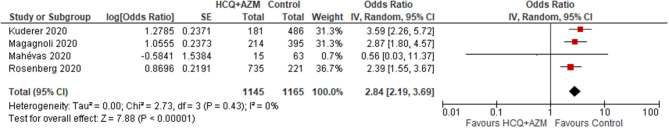
All-cause mortality (HCQ + AZM vs. control; observational studies).

Four studies (*N* = 2,310) reported a significant increase in the mortality rate in the HCQ plus AZM group compared to the control group (OR 2.84; 95% CI 2.19–3.69; *p* < 0.001) (19, 21, 25, 27) (Figure 3). Another study used Azithromycin in treatment but did not provide separate data (19).

##### HCQ vs. HCQ plus AZM

Five studies (*N* = 1,988) reported mortality rate, and found a significant decrease in HCQ group (OR 0.7; 95% CI 0.54–0.9; *p* = 0.006; *I*^2^ = 0%) (19, 21, 22, 25, 27).

##### High-dose vs. low-dose CQ

One RCT (*N* = 81) found a significantly higher mortality rate in the high-dose group (OR 3.63; 95% CI 1.24–10.58; *p* = 0.02) (Supplementary Figure 2) (18).

#### Secondary Outcomes

Details have been provided in Table 2. A majority of the outcome measures favored the Control group (i.e., the Control was better than HCQ±AZM), and these were the occurrence of adverse events [any events (Supplementary Figure 3), or only cardiac events, or only vomiting], development of severe disease, and duration of hospitalization. Those favored HCQ group (i.e., HCQ±AZM was better than Control) were resolution of cough, proportion of patients with negative COVID-19 PCR after days 5, 10, and 14, proportion of patients with improved radiological features after day 5, change in IL-6 level (pg/mL), and change in total leukocyte count (/cumm). The outcomes that favored HCQ over HCQ plus AZM were mortality rate and the development of severe disease. Contrary to common belief, no difference between HCQ and HCQ plus AZM was found for any type of adverse cardiac events.

**Table 2:** Secondary outcome measures from the included studies.

**Name of outcome**	**No of trials (Reference)**	**Sample size**	**Effect estimate**	***P*-value**
**CLINICAL TRIALS (RANDOMIZED, AND NON-RANDOMIZED)**				
**Hydroxy-chloroquine (HCQ)/chloroquine (CQ) vs. control**				
Time to alleviation of clinical symptoms (d)				
Fever	2 (14, 17)	92	MD 0.21; 95% CI −2.95 to 3.37	0.9
Cough	1 (17)	62	MD −1.1; 95% CI (−1.86 to −0.34)	0.005^*^
Clinical recovery	1 (15)	119	Could not be pooled	0.96
Time to negative RT- PCR results (d)	2 (14, 15)	180	MD 1.55; 95% CI −0.7 to 3.79	0.18
Escalation of respiratory support (including MV)	1 (13)	42	OR 4.92; 95% CI 0.24 to 101.66	0.3
Development of severe disease	1 (17)	62	OR 0.1; 95% CI 0.0 to 1.88	0.12
Proportion with clinical recovery after day 28	1 (15)	150	OR 0.75; 95% CI 0.39 to 1.46	0.34
Proportion with negative RT- PCR				
After day 3	2 (13, 15)	180	OR 1.02; 95% CI 0.16 to 6.6	0.98
After day 5	1 (13)	30	OR 9.33; 95% CI 1.51 to 57.65	0.02^*^
After day 7	3 (14–16)	202	OR 0.65; 95% CI 0.36 to 1.17	0.15
After day 10	1 (15)	150	OR 0.73; 95% CI 0.37 to 1.47	0.38
After day 14	3 (14–16)	202	OR 0.98; 95% CI 0.44 to 2.15	0.95
After day 21	1 (15)	150	OR 1.49; 95% CI 0.62 to 3.61	0.37
After day 28	1 (15)	150	Not pooled (event NE in HCQ group)	
Proportion with improved radiological features				
After day 3	1 (14)	30	OR 0.57; 95% CI 0.13 to 2.5	0.46
After day 5	1 (17)	62	OR 3.43; 95% CI 1.1 to 10.7	0.03^*^
After day 14	1 (14)	30	All patients (HCQ and control group) improved	
Adverse events				
Any	4 (14–17)	263	OR 3.35; 95% CI 1.58 to 7.13	0.002^*^
Serious	1 (15)	150	OR 5.88; 95% CI 0.28 to 124.5	0.26
Vomiting	2 (15, 16)	172	OR 8.67; 95% CI 1.32 to 56.99	0.02^*^
Abdominal complaints	2 (15, 16)	172	OR 0.77; 95% CI 0.12 to 5.11	0.79
Diarrhea	3 (14–16)	202	OR 2.45; 95% CI 0.25 to 24.18	0.44
Transamnitis	2 (14, 15)	180	OR 1.74; 95% CI 0.2 to 14.78	0.61
Kidney injury	2 (14, 15)	180	OR 1.06; 95% CI 0.1 to 11.3	0.96
**Hydroxy-chloroquine (HCQ) and azithromycin (AZM) vs. control**				
Proportion of patients with negative RT-PCR				
After day 3	1 (13)	22	OR 15.0; 95% CI 1.32 to 169.89	0.03^*^
After day 5	1 (13)	22	OR 0.45; 95% CI 0.02 to 10.67	0.62
**High-dose vs. low-dose chloroquine (CQ)**				
Proportion of patients with negative RT-PCR				
After day 3	1 (18)	27	No separate data (six patients negative)	NE
Adverse events	1 (18)	81	OR 2.27; 95% CI 1.14 to 4.49	0.02^*^
**Name of outcome**	**No of studies (Reference)**	**Sample size**	**Effect estimate**	***P*****-value**
**OBSERVATIONAL STUDIES**				
**Hydroxy-chloroquine (HCQ) or Chloroquine (CQ) vs. control**				
Escalation of respiratory support (including MV)	5 (19–21, 25, 27)	3,247	OR 2.04; 95% CI 0.99 to 4.18	0.05
Development of severe disease	3 (19, 21, 24)	1,038	OR 1.12; 95% CI 0.51 to 2.46	0.77
Duration of hospitalization (d)	5 (21, 23–25, 29)	1,858	MD 2.17; 95% CI 0.21 to 4.13	0.03^*^
Time to negative RT- PCR results (d)	2 (24, 28)	407	MD 1.14; 95% CI −11.98 to 14.26	0.86
Proportion of patients with negative RT- PCR				
After day 10	1 (24)	373	OR 7.86; 95% CI 4.4 to 14.04	<0.001^*^
After day 14	2 (24, 28)	407	OR 6.37; 95% CI 3.01 to 13.48	<0.001^*^
Proportion with improved radiological features				
After day 10	1 (24)	71	OR 1.13; 95% CI 0.38 to 3.3	0.83
After day 14	1 (24)	71	OR 0.88; 95% CI 0.32 to 2.46	0.81
Effect on hematological parameters				
Change in IL-6 level (pg/mL)	1 (23)	550	MD −20.64; 95% CI −26.24 to −15.04	<0.001^*^
Change in CRP level (mg/L)	1 (28)	34	MD −4.95; 95% CI −34.17 to 24.27	0.74
Change in total leukocyte count (/cumm)	1 (28)	34	MD −1247.7; 95% CI −2356.6 to −138.7	0.03^*^
Change in total lymphocyte count (/cumm)	1 (28)	34	MD −190.75; 95% CI −998.12 to 616.62	0.64
Change in serum ferritin (μg/L)	1 (28)	34	MD −165.97; 95% CI −680.53 to 348.59	0.53
Adverse events				
Any	1 (24)	373	OR 0.77; 95% CI 0.49 to 1.2	0.25
Abnormal ECG	2 (19, 21)	665	OR 4.17; 95% CI 0.63 to 27.58	0.14
Arrhythmia	2 (21, 22)	693	OR 1.44; 95% CI 0.87 to 2.39	0.16
QT prolongation	3 (19, 21, 22)	866	OR 1.8; 95% CI 0.79 to 4.11	0.16
Cardiac arrest	1 (21)	492	OR 2.17; 95% CI 1.16 to 4.07	0.02^*^
Diarrhea	2 (21, 28)	865	OR 0.8; 95% CI 0.34 to 1.85	0.60
Hypoglycemia	1 (21)	492	OR 1.23; 95% CI 0.43 to 3.51	0.70
**Hydroxy-chloroquine (HCQ) and azithromycin (AZM) vs. control**				
Escalation of respiratory support (including MV)	4 (19, 21, 25, 27)	2,294	OR 2.18; 95% CI 0.63 to 7.57	0.22
Development of severe disease	1 (21)	492	OR 3.19; 95% CI 2.07 to 4.91	<0.001^*^
Duration of hospitalization (d)	2 (21, 25)	1,180	MD 3.6; 95% CI 1.6 to 5.61	<0.001^*^
Adverse events				
Abnormal ECG	1 (21)	492	OR 2.28; 95% CI 1.51 to 3.44	<0.001^*^
QT prolongation	1 (21)	492	OR 1.98; 95% CI 1.08 to 3.63	0.03^*^
Arrhythmia	1 (21)	492	OR 2.21; 95% CI 1.38 to 3.52	<0.001^*^
Cardiac arrest	1 (21)	492	OR 2.52; 95% CI 1.44 to 4.42	0.001^*^
Diarrhea	1 (21)	492	OR 1.68; 95% CI 0.96 to 2.92	0.07
Hypoglycemia	1 (21)	492	OR 1.26; 95% CI 0.51 to 3.12	0.61
**Hydroxy-chloroquine (HCQ) vs. HCQ and azithromycin (AZM)**				
Escalation of respiratory support (including MV)	4 (19, 21, 25, 27)	1,730	OR 0.66; 95% CI 0.41 to 1.05	0.08
Development of severe disease	1 (21)	1,006	OR 0.53; 95% CI 0.38 to 0.75	<0.001^*^
Duration of hospitalization (d)	1 (25)	262	MD −1.0; 95% CI −2.46 to 0.46	0.18
Adverse events				
Abnormal ECG	1 (21)	1,006	OR 1.01; 95% CI 0.74 to 1.38	0.94
QT prolongation	2 (21, 22)	1,207	OR 1.28; 95% CI 0.88 to 1.87	0.20
Arrhythmia	2 (21, 22)	1,207	OR 0.74; 95% CI 0.52 to 1.06	0.10
Cardiac arrest	1 (21)	1,006	OR 0.86; 95% CI 0.58 to 1.29	0.46
Diarrhea	1 (21)	1,006	OR 0.68; 95% CI 0.41 to 1.1	0.12
Hypoglycemia	1 (21)	1,006	OR 0.98; 95% CI 0.45 to 2.12	0.95

*OR, Odds ratio; MD, Mean difference; CI, Confidence interval; NE, Not estimable; PCR, Polymerase chain reaction; MV, Mechanical ventilation; ECG, Electrocardiogram*.

*P < 0.05 significant.

### Publication Bias

The funnel plot was asymmetrical showing publication bias (Supplementary Figure 4). The reasons for publication bias were heterogeneity among studies, poor methodological design, and selective outcome reporting.

### Grade of Evidence

The evidence generated was of “very low quality” for all the outcomes (primary and secondary). A detailed analysis of the summary of evidence is provided in Table 3.

**Table 3:** GRADE evidence (anti-malarial drugs ± azithromycin vs. standard of care for patients with COVID-19 infection).

**Outcomes**	**No of Participants (studies)**	**Quality of the evidence (GRADE)**	**Relative effect (95% CI)**	**Anticipated absolute effects**	
				**Risk with standard of care**	**Risk difference with anti-malarial drugs (95% CI)**
**Primary outcome measures (HCQ or HCQ+AZM vs. control/supportive care and high vs. low-dose CQ)**					
All-cause mortality (HCQ vs. control)	5,944 (eight Observational studies)	⊕⊖⊖⊖ **VERY LOW**^a^^,^ ^b^^,^ ^c^^,^ ^d^^,^ ^e^ due to risk of bias, inconsistency, indirectness, imprecision, publication bias	**OR 0.87** (0.46–1.64)	**Study population**	
				**202 per 1,000**	**22 fewer per 1,000** (from 98 fewer to 91 more)
All-cause mortality (HCQ vs. control)	42 (one Non-RCT)	⊕⊖⊖⊖ **VERY LOW**^d^^,^ ^f^^,^ ^g^^,^ ^h^^,^ ^i^^,^ ^j^ due to risk of bias, inconsistency, indirectness, imprecision	**OR 1.94** (0.07–50.57)	**Study population**	
				**Not estimable**	**Not estimable (“0” event in control/standard of care group)**
All-cause mortality (HCQ+azithromycin vs. control)	2,310 (four Observational studies)	⊕⊖⊖⊖ **VERY LOW**^a^^,^ ^b^^,^ ^c^^,^ ^i^ due to risk of bias, inconsistency, indirectness, publication bias	**OR 2.84 (2.19–3.69)**	**Study population**	
				**94 per 1,000**	**133 more per 1,000** (from 91 more to 182 more)
All-cause mortality (HCQ vs. HCQ+azithromycin)	1,988 (five Observational studies)	⊕⊖⊖⊖ **VERY LOW**^a^^,^ ^c^^,^ ^d^^,^ ^f^^,^ ^h^ due to risk of bias, inconsistency, indirectness, imprecision, publication bias	**OR 0.7** (0.54–0.9)	**Study population**	
				**226 per 1,000**	**46 fewer per 1,000** (from 2 fewer to 83 fewer)
All-cause mortality (CQ: high-dose vs. low-dose)	81 (one RCT)	⊕⊖⊖⊖ **VERY LOW**^d^^,^ ^f^^,^ ^i^^,^ ^k^ due to risk of bias, inconsistency, indirectness, imprecision	**OR 3.63** (1.24–10.58)	**Study population**	
				**150 per 1,000**	**240 more per 1,000** (from 30 more to 501 more)
Secondary outcome measures (HCQ vs. control/supportive care)^**^					
Duration of hospitalization (day)	1,858 (five Observational studies)	⊕⊖⊖⊖ **VERY LOW**^a^^,^ ^c^^,^ ^e^^,^ ^f^^,^ ^h^ due to risk of bias, inconsistency, indirectness, publication bias	**MD 2.17** (0.21–4.13)		The mean duration of hospitalization (day) in the intervention groups was **2.17 higher** (0.21 to 4.13 higher)
Any adverse events	264 (four RCTs)	⊕⊖⊖⊖ **VERY LOW**^c^^,^ ^f^^,^ ^g^^,^ ^h^^,^ ^j^ due to risk of bias, inconsistency, indirectness, publication bias	**OR 3.35** (1.58–7.13)	**Study population**	
				**145 per 1,000**	**217 more per 1,000** (from 66 more to 402 more)
Proportions with negative COVID-19 PCR after day 14	407 (two Observational studies)	⊕⊖⊖⊖ **VERY LOW**^a^^,^ ^c^^,^ ^f^^,^ ^h^ due to risk of bias, indirectness, inconsistency, publication bias	**OR 6.37** (3.01–13.48)	**Study population**	
				**594 per 1,000**	**309 more per 1,000** (from 221 more to 358 more)

*The basis for the **assumed risk** (e.g., the median control group risk across studies) is provided in footnotes. The **corresponding risk** (and its 95% confidence interval) is based on the assumed risk in the comparison group and the **relative effect** of the intervention (and its 95% CI). CI, Confidence interval; OR, Odds ratio; MD, Mean difference.

** *Secondary outcomes reporting pooled results from minimum two studies with significant difference between groups are reported here*.

GRADE Working Group grades of evidence **High quality:** Further research is very unlikely to change our confidence in the estimate of effect. **Moderate quality:** Further research is likely to have an important impact on our confidence in the estimate of effect and may change the estimate. **Low quality:** Further research is very likely to have an important impact on our confidence in the estimate of effect and is likely to change the estimate. **Very low quality:** We are very uncertain about the estimate.

a *case-control study*.

b *Inhomogeneous population with many being >65 years and male (not matched for age and sex confounders)*.

c *Patients in both the groups also received additional treatment which might influence the outcome, but not clearly defined*.

d *The 95% CI around the pooled effect is wide and different in the included studies. The 95% CI includes no effect*.

e *Being published on pre-print server and not in a peer-reviewed journal*.

f *Both the groups were not homogenous considering the age and sex of the participants*.

g *Open label trials*.

h *Different dose schedule of intervention used*.

i *Single study*.

j *Single country data*.

k Though described as double-blinded, blinding of investigators, participants, and outcome accessor unclear. Allocation concealment also unclear. ^k^One trial is open label. ^l^Significant statistical heterogeneity. ^m^Two trials are open label and one double-blinded (but this trial has unclear blinding and allocation concealment). ^n^Wider 95% CI. ^o^Developed country setting data that cannot be apply to developing country setting.

## Discussion

### Summary of Evidence

After an extensive search of the literature, we included 17 studies with data of 8,071 participants. Compared to control, HCQ alone (not HCQ+AZM combination) has no significant effect on mortality or risk of adverse cardiac events. The evidence for all the outcomes was of “very low quality.”

The high mortality and increased risk of adverse events with anti-malarial drugs noted by some studies may be overestimated because of the inclusion of an older population with underlying co-morbidities (including cardiac conditions) and simultaneous use of other cardiotoxic drugs (e.g., Azithromycin, and Oseltamivir). The same may be difficult to know during the current pandemic as there is no definitive treatment, and healthcare professionals all over the world want to administer these experimental drugs with the hope of saving some lives. The use of HCQ+AZM has drawn attention, and there are differences in opinion regarding use of this combination. Compared to control, HCQ+AZM combination was found to increase the mortality rate significantly, in contrast to HCQ alone. Compared to HCQ+AZM combination, HCQ alone was significantly decreasing the mortality rate. These indirect evidences suggest that HCQ+AZM might increase the mortality rate, and caution should be exercised while using this combination in vulnerable population (e.g., those with advanced age, underlying cardiac conditions, and those receiving medication with cardiac side-effects, as noted in the included studies).

It has to be kept in mind that, the anti-viral action of anti-malarial drugs against COVID-19 is still largely unknown (49, 50). An acute systemic inflammatory reaction/cytokine storm (besides the viral infection itself) is the hallmark of COVID-19 infection (51). This reaction, once well-established, can cause rapid disease progression leading to death (52, 53). However, except for three studies (15, 23, 28), no studies have reported the effect of anti-malarial drugs on the inflammatory markers and blood counts (lymphocyte, neutrophil). As supportive treatments were not uniform across the included studies, one may argue that simultaneous use of other drugs (anti-viral drugs, and/or interferon-α) as a part of supportive treatment might have confounded (increased or decreased) the efficacy of the anti-malarial drugs (15). This possibility, however, seems less likely, as few studies have found no difference after excluding patients receiving these drugs (15).

An interesting observation was that, studies from USA showed a significantly increased risk of mortality compared to those from outside USA. The same could be explained by the following points in the USA study cohort: inclusion of a higher proportion of patients with severe or critical illness, advanced age, and co-morbidities. Among the included studies in the present review, marked variation (high heterogeneity) was noted in the age group (in the clinical trials majority were ≤ 50 yr of age, whereas, in the observational studies majority were ≥60 yr of age), severity of COVID-19 illness (around 72% of participants in the clinical trials were having mild and moderate illness, whereas <40% of the participants in the observational studies exhibited mild and moderate illness), and inclusion of patients with co-morbidities (diabetes, cardio-vascular disease, chronic lung disease, etc.) among the study cohorts. We could not, however, carry out sub-group analyses as per severity illness because of paucity of data. Except for the severity of COVID-19 illness, the remaining two characteristics (age group and inclusion of patients with co-morbidities) of the study cohort could increase mortality that is independent of the effect of CQ/HCQ (±Azithromycin). This emphasizes the role of randomized double-blind trials in establishing the actual efficacy (if any) of anti-malarial drugs, as the chance of selection bias would be very low, and the groups would be comparable. The dose schedule of CQ was nearly uniform; however, the dose schedule of HCQ varied widely among the studies (except for one large study, the cumulative dose was equal or higher than the recommended schedule in the remaining studies). There was, however, no difference in the mortality rate. The median time from onset of symptom to admission or treatment initiation was nearly ≤ 8 days in all but two studies, and, apart from one study, others used CQ/HCQ within 48 h of admission/hospitalization (not symptom onset). There was no significant difference in the mortality rate between exposure/interventions and controls in these sub-groups. This might be due to the fact that starting anti-viral drugs (including HCQ/CQ) after 48 h of symptom onset might not be beneficial as the golden window for antiviral treatment (e.g., in influenza) is lost (48). This is difficult in a hospitalized setting (may be possible in outpatient or community setting); however, one RCT could able to use it within 48 h of symptom onset (found a significantly shorter time to clinical recovery and pneumonia resolution without any mortality) (17).

### Limitations

The studies were variable in many aspects (blinding of participants and outcome assessors, patient selection, severity of illness, dose schedule of the anti-malarial drugs, timing of administration, measurement of inflammatory markers and effect of the drugs on these markers, outcome definition, and measurements). We could not determine the effect of anti-malarial drugs in Covid-19 infection in pediatric and adolescent population. As there were few studies, results from all the secondary outcomes could not be pooled.

### Future Areas of Research

Future clinical trials should include good quality RCTs with adequate sample size, should ideally be multi-centric, and should focus on the variability noted in the present review. Pediatric and adolescent population also need to be included in the ongoing studies to guide recommendation in this group of patients. Both CQ/HCQ should also be evaluated in non-hospitalized patients with COVID-19 infection.

## Conclusions

As very low quality evidence suggests an increased risk of mortality and adverse event with HCQ plus Azithromycin combination (not HCQ alone); caution should be exercised while prescribing this combination for treatment of hospitalized adults with COVID-19 infection. Multi-centric RCTs (including both hospitalized and non-hospitalized patients) of a good quality are required for any firm recommendation to be made during the ongoing pandemic.

## Data Availability Statement

All datasets generated for this study are included in the article/Supplementary Material.

## Author Contributions

RD developed the concept, abstracted data from papers, managed data for the review, performed the statistical analysis, interpreted data, made statistical inferences, wrote the review, and serves as guarantor for the review. NisJ, ND, NikJ, and SN abstracted data from papers, managed data for the review, and wrote the review. JS developed the concept, performed the statistical analysis and made inferences, and wrote the review. RD and JS jointly act as guarantors. All the authors have approved the version to be published.

## Conflict of Interest

The authors declare that the research was conducted in the absence of any commercial or financial relationships that could be construed as a potential conflict of interest.

## Supplementary Material

Supplementary Figure 1 All-cause mortality (HCQ vs. control; Non-RCT).

Supplementary Figure 2 All-cause mortality (High-dose vs. low-dose CQ; RCT).

Supplementary Figure 3 Any adverse events (HCQ vs. control; RCTs).

Supplementary Figure 4 Funnel plot (primary outcome data from observational studies).
