# Supplementary figures and images for: Efficacy and Safety of Anti-malarial Drugs (Chloroquine and Hydroxy-Chloroquine) in Treatment of COVID-19 Infection: A Systematic Review and Meta-Analysis
Source: Front Med (Lausanne). 2020 Jul 29;7:482. doi: 10.3389/fmed.2020.00482 (PMC7403461; doi:10.3389/fmed.2020.00482)

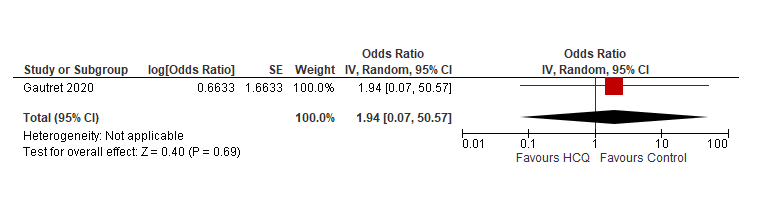

Supplement: Supplementary Figure 1 — All-cause mortality (HCQ vs. control; Non-RCT). [file Image_1.tif]

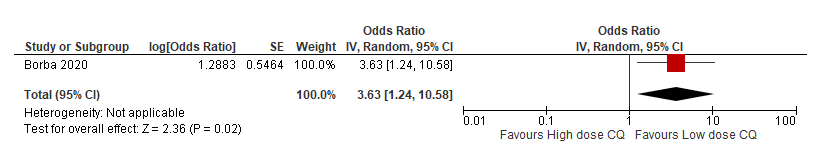

Supplement: Supplementary Figure 2 — All-cause mortality (High-dose vs. low-dose CQ; RCT). [file Image_2.TIF]

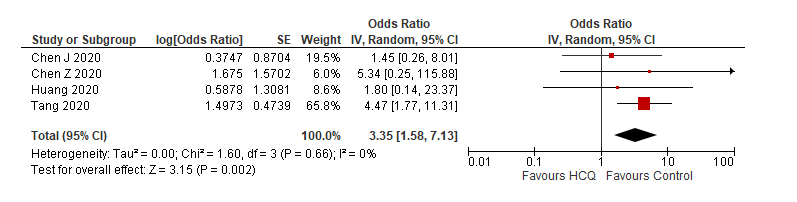

Supplement: Supplementary Figure 3 — Any adverse events (HCQ vs. control; RCTs). [file Image_3.TIF]

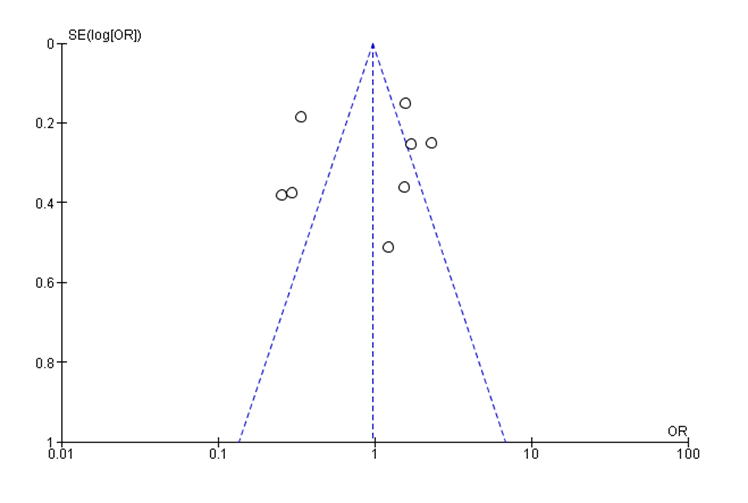

Supplement: Supplementary Figure 4 — Funnel plot (primary outcome data from observational studies). [file Image_4.TIF]
