# Supplementary material for: Efficacy and Safety of Anti-malarial Drugs (Chloroquine and Hydroxy-Chloroquine) in Treatment of COVID-19 Infection: A Systematic Review and Meta-Analysis
Source: Front Med (Lausanne). 2020 Jul 29;7:482. doi: 10.3389/fmed.2020.00482 (PMC7403461; doi:10.3389/fmed.2020.00482)
Supplement: Supplementary file 5 [file Data_Sheet_1.docx]

**Appendix 1**

| **Medline Search** | |
| --- | --- |
| **Query** | **Items** |
| Search ((((((covid 19) OR ("COVID-19" [Supplementary Concept] OR "severe acute respiratory syndrome coronavirus 2" [Supplementary Concept])) OR ((((((((2019-nCoV) OR Wuhan coronavirus) OR SARS-CoV-2) OR 2019 novel coronavirus) OR COVID-19 virus) OR coronavirus disease 2019 virus) OR Wuhan seafood market pneumonia virus) OR COVID19 virus))) AND ((((hydroxychloroquine) OR (( "Hydroxychloroquine/administration and dosage"[Mesh] OR "Hydroxychloroquine/adverse effects"[Mesh] OR "Hydroxychloroquine/therapeutic use"[Mesh] )))) OR ((((((((((((((((((Plaquenil) OR Oxychlorochin) OR Oxychloroquine) OR Hydroxychloroquine Sulfate) OR Hydroxychloroquine Sulfate (1:1) Salt) OR Aminoquinolines) OR Chlorochin) OR Chingamin) OR Khingamin) OR Nivaquine) OR Chloroquine Sulfate) OR Sulfate, Chloroquine) OR Chloroquine Sulphate) OR Sulphate, Chloroquine) OR Aralen) OR Arequin) OR Arechine) OR Chloroquine)))) NOT ((animals) NOT humans) | 626 |
| Search (animals) NOT humans | 4643733 |
| Search humans | 18524730 |
| Search animals | 6681571 |
| Search ((((covid 19) OR ("COVID-19" [Supplementary Concept] OR "severe acute respiratory syndrome coronavirus 2" [Supplementary Concept])) OR ((((((((2019-nCoV) OR Wuhan coronavirus) OR SARS-CoV-2) OR 2019 novel coronavirus) OR COVID-19 virus) OR coronavirus disease 2019 virus) OR Wuhan seafood market pneumonia virus) OR COVID19 virus))) AND ((((hydroxychloroquine) OR (( "Hydroxychloroquine/administration and dosage"[Mesh] OR "Hydroxychloroquine/adverse effects"[Mesh] OR "Hydroxychloroquine/therapeutic use"[Mesh] )))) OR ((((((((((((((((((Plaquenil) OR Oxychlorochin) OR Oxychloroquine) OR Hydroxychloroquine Sulfate) OR Hydroxychloroquine Sulfate (1:1) Salt) OR Aminoquinolines) OR Chlorochin) OR Chingamin) OR Khingamin) OR Nivaquine) OR Chloroquine Sulfate) OR Sulfate, Chloroquine) OR Chloroquine Sulphate) OR Sulphate, Chloroquine) OR Aralen) OR Arequin) OR Arechine) OR Chloroquine)) | 73 |
| Search (((hydroxychloroquine) OR (( "Hydroxychloroquine/administration and dosage"[Mesh] OR "Hydroxychloroquine/adverse effects"[Mesh] OR "Hydroxychloroquine/therapeutic use"[Mesh] )))) OR ((((((((((((((((((Plaquenil) OR Oxychlorochin) OR Oxychloroquine) OR Hydroxychloroquine Sulfate) OR Hydroxychloroquine Sulfate (1:1) Salt) OR Aminoquinolines) OR Chlorochin) OR Chingamin) OR Khingamin) OR Nivaquine) OR Chloroquine Sulfate) OR Sulfate, Chloroquine) OR Chloroquine Sulphate) OR Sulphate, Chloroquine) OR Aralen) OR Arequin) OR Arechine) OR Chloroquine) | 33458 |
| Search (((((((((((((((((Plaquenil) OR Oxychlorochin) OR Oxychloroquine) OR Hydroxychloroquine Sulfate) OR Hydroxychloroquine Sulfate (1:1) Salt) OR Aminoquinolines) OR Chlorochin) OR Chingamin) OR Khingamin) OR Nivaquine) OR Chloroquine Sulfate) OR Sulfate, Chloroquine) OR Chloroquine Sulphate) OR Sulphate, Chloroquine) OR Aralen) OR Arequin) OR Arechine) OR Chloroquine | 31786 |
| Search ((covid 19) OR ("COVID-19" [Supplementary Concept] OR "severe acute respiratory syndrome coronavirus 2" [Supplementary Concept])) OR ((((((((2019-nCoV) OR Wuhan coronavirus) OR SARS-CoV-2) OR 2019 novel coronavirus) OR COVID-19 virus) OR coronavirus disease 2019 virus) OR Wuhan seafood market pneumonia virus) OR COVID19 virus) | 3863 |
| Search (((((((2019-nCoV) OR Wuhan coronavirus) OR SARS-CoV-2) OR 2019 novel coronavirus) OR COVID-19 virus) OR coronavirus disease 2019 virus) OR Wuhan seafood market pneumonia virus) OR COVID19 virus | 1906 |
| Search "COVID-19" [Supplementary Concept] OR "severe acute respiratory syndrome coronavirus 2" [Supplementary Concept] | 819 |
| Search covid 19 | 3814 |
| Search (hydroxychloroquine) OR (( "Hydroxychloroquine/administration and dosage"[Mesh] OR "Hydroxychloroquine/adverse effects"[Mesh] OR "Hydroxychloroquine/therapeutic use"[Mesh] )) | 4966 |
| Search ( "Hydroxychloroquine/administration and dosage"[Mesh] OR "Hydroxychloroquine/adverse effects"[Mesh] OR "Hydroxychloroquine/therapeutic use"[Mesh] ) | 2587 |
| Search hydroxychloroquine | 4966 |

| **Embase Search** | | |
| --- | --- | --- |
| **No.** | **Query** | Items |
| #1 | ('covid 19'/exp OR 'covid 19' OR 'sars       coronavirus 2'/exp OR 'sars coronavirus 2') AND       ('hydroxychloroquine sulfate'/exp OR       'chloroquine'/exp OR 'aminoquinoline       derivative'/exp OR 'aminoquinoline derivative' OR       'aminoquinolines') | 749 |
