# Supplementary material for: Efficacy and Safety of Anti-malarial Drugs (Chloroquine and Hydroxy-Chloroquine) in Treatment of COVID-19 Infection: A Systematic Review and Meta-Analysis
Source: Front Med (Lausanne). 2020 Jul 29;7:482. doi: 10.3389/fmed.2020.00482 (PMC7403461; doi:10.3389/fmed.2020.00482)
Supplement: Supplementary file 6 [file Data_Sheet_2.docx]

| **Risk of bas: Randomized controlled clinical trials (Cochrane risk of bias tool)** | | | | | | |
| --- | --- | --- | --- | --- | --- | --- |
| **Study(Reference)** | **Random sequence generation** | **Allocation concealment** | **Blinding of personals & participants** | **Blinding of outcome assessors** | **Selective reporting** | **Missing data** |
| Chen 2020 (14) | Unclear | Unclear | High | High | Low | Low |
|  | Reason: Not mentioned | Reason: Not mentioned | Reason: No (open label) | Reason: No (open label). | Reason: No | Reason: No attrition |
| Tang 2020 (15) | Low | Low | High | High | Low | Low |
|  | Reason: Stratified according to the disease severity (mild/moderate or severe) and were then randomly assigned (in a 1:1 ratio) to receive interventions. Patients were enrolled by the site investigator. The statistician performed the randomization; equal numbers of cards with each group assignment number randomly generated by computer | Reason: Sequentially numbered envelopes that were opened as the patients were enrolled. | Reason: No (open label) | Reason: No (open label) | Reason: No | Reason: 6% attrition rate in HCQ group. Analyzed as ITT (intention-to-treat) |
| Huang 2020 (16) | Unclear | Unclear | Unclear | Unclear | Unclear | Low |
|  | Reason: Not mentioned | Reason: Not mentioned | Reason: Not mentioned | Reason: Not mentioned | Reason: Not mentioned | Reason: Not mentioned |
| Chen 2020 (17) | Unclear | Low | Unclear | Unclear | Low | Low |
|  | Reason: Significant protocol deviation (in protocol mentioned as computer generated block randomization [4 blocks of 110 participants per block, total 440 patients], but actual trial reported data of 62 patients. | Reason: Significant protocol deviation and no clear mention about 62 included patients. | Reason: Not mentioned | Reason: Not mentioned | Reason: Not mentioned | Reason: No attrition. |
| Borba 2020 (18) | Low | Low | Unclear | Unclear | High | Low |
|  | Reason: Electronically generated randomization list was prepared by an independent statistician, with four blocks of 110 participants per block. This randomization list associated each patient's study number with an opaque surface hiding the treatment group designation. The list was accessible only to non-blinded pharmacists in the study, in an attempt to minimize observation bias | Reason: Randomization list associated each patient's study number with an opaque surface hiding the treatment group designation | Reason: Details not discussed | Reason: Details not discussed | Reason: Other important outcomes not mentioned (though mentioned in methods & protocol) | Reason: No attrition |

**Appendix 2**

| **Risk of bias: Non-randomized controlled clinical trials (ROBINS-I tool)** | | | | | | | |
| --- | --- | --- | --- | --- | --- | --- | --- |
| **Study (Reference)** | **Bias due to confounding** | **Bias in selection of participants** | **Bias in classification of interventions** | **Bias due to deviations from intended interventions** | **Bias due to missing data** | **Bias in measurement of outcomes** | **Bias in selection of the reported result** |
| Goutret 2020 (13) | Serious | Serious | Low | Critical | Critical | Moderate | Serious |
|  | Reason: Baseline imbalance in the age of participants (higher and with more co-morbidities in intervention group) creating an imbalance) | Reason: Two groups were recruited at different hospitals with possible different standard of care. The participants who refused consent or not eligible for the drug served as control | Reason: Prospective study | Reason: 6 (30%) patients in HCQ group received AZT | Reason: 6 (23%) patients in HCQ group loss to follow-up. No intention-to-treat analysis | Reason: Unclear if the outcome assessors (laboratory personnel) were blinded | Reason: Planned outcomes were not reported. Time point reported in the study (6-day) was not planned in protocol |

| **Risk of bias: Observational studies (New-Castle Ottawa Scale)** | | | | | | | | | |
| --- | --- | --- | --- | --- | --- | --- | --- | --- | --- |
| **Study (Reference)** | **Selection** | | | | **Comparability** | **Exposure** | | | **Total** |
|  | Is the case definition adequate? | Representativeness of the cases | Selection of Controls | Definition of Controls | Comparability of cases and controls on the basis of the design or analysis | Ascertainment of exposure | Same method of ascertainment for cases and controls | Non-Response rate |  |
| Mahévas 2020 (19) | **__ | | | | * | *** | | | ****** |
| Geleris 2020 (20) | **__ | | | | * | *** | | | ****** |
| Rosenberg 2020 (21) | **__ | | | | * | *** | | | ****** |
| Saleh 2020 (22) | *___ | | | | * | ** | | | **** |
| Yu 2020 (23) | *___ | | | | * | *** | | | ***** |
| Huang 2020 (24) | **__ | | | | * | *** | | | ***** |
| Magagnoli 2020 (25) | *___ | | | | * | ** | | | **** |
| Ayerbe 2020 (26) | **__ | | | | * | *** | | | ***** |
| Kuderer 2020 (27) | _*** | | | | * | *** | | | ******* |
| Mallat 2020 (28) | _*__ | | | | * | *** | | | ***** |
| Magagnoli 2020 (29) | _*__ | | | | * | *** | | | ***** |

Footnote: This form has been designed to assess case control studies based on: (1) Selection of subjects (maximum of 4 stars were given one against each item); (2) Selection of controls (maximum of 2 stars were given) and (3) Assessment of exposure (maximum of 3 stars were given one against each numbered item)
