# Supplementary material for: Efficacy and Safety of Anti-malarial Drugs (Chloroquine and Hydroxy-Chloroquine) in Treatment of COVID-19 Infection: A Systematic Review and Meta-Analysis
Source: Front Med (Lausanne). 2020 Jul 29;7:482. doi: 10.3389/fmed.2020.00482 (PMC7403461; doi:10.3389/fmed.2020.00482)
Supplement: Supplementary file 7 [file Table_1.doc]

**Supplementary Table 1: Characteristics of studies published in pre-print server (not peer-reviewed)**

| **Observational studies** | | | | | |
| --- | --- | --- | --- | --- | --- |
| **Study author (Reference)** | **Setting, Country** | **No of participants (disease severity)** | **Intervention and comparator details, supportive treatment** | **Outcomes measured** | **Findings** |
| Chen 2020 (30) | Inpatient,  China  (single center) | 284  (all severity) | Exposure group: Chloroquine (CQ) for 7 days.  Control group: No CQ.  Supportive treatment: Antibiotics (75.0%), oxygen (64.8%), antivirals [oseltamivir (12.7%), lopinavir/ritonavir (27.5%), & arbidol (24.3%)], corticosteroid (10.9%), immunoglobulin (3.2%) & ribavirin (0.7%). | Time to virological cure clearance, duration of hospital stay | The use of antiviral drugs (including CQ) does not enhance viral  clearance. |
| Feng 2020 (31) | Inpatient,  China  (multi-center) | 50  (all severity) | Exposure group: Chloroquine (CQ) for 7 days.  Control group: No CQ.  Supportive treatment: Antivirals (Arbidol and Lopinavir/ritonavir (96%), Interferon α (36%), and intravenous antibiotics (44%). | Progression to severe pneumonia, effect on hematological & inflammatory parameters, change in CT scan findings, and adverse events | Though none of the current antiviral and immunoregulation therapy showed benefit in preventing COVID-19 progression, Chloroquine deserved further investigation. |
| Shabrawishi 2020 (32) | Inpatient,  Saudi-Arabia (Single center) | 93  (mild & moderate cases) | Exposure (A) group: A1 – CQ /HCQ only, A2 – CQ/HCQ + Azithromycin, and A3 – CQ/HCQ + antiviral drugs).  Control (B) group: No CQ/HCQ ± AZM.  Supportive treatment: No clear. | Achieving virological cure ≤5 days from the start of the intervention & ≤12 days from the diagnose, respectively | No significant different in time to virological cure between intervention group and control group. |
